# Unlocking the Potential of Paper Mulberry Powder in Cherry Valley Ducks: Impacts on Growth, Serum Biochemistry, and Cecum Microbiome

**DOI:** 10.3390/ani15111602

**Published:** 2025-05-30

**Authors:** Yi Xiong, Chu Tang, Xuekai Wang, Yongsheng Wang, Fuyu Yang

**Affiliations:** 1 Frontier Technology Research Institute of China Agricultural University in Shenzhen, Shenzhen 518000, China; xiongleslie@126.com; 2 College of Grassland Science and Technology, China Agricultural University, Beijing 100193, China; 3 Agricultural Genomics Institute at Shenzhen, Chinese Academy of Agricultural Sciences, Shenzhen 518000, China

**Keywords:** paper mulberry powder, Cherry Valley duck, growth performance, serum biochemistry, cecum microbiome

## Abstract

This study investigates the effects of incorporating paper mulberry (*Broussonetia papyrifera* L.) powder into the diets of Cherry Valley ducks on growth performance, serum biochemistry, and the gut microbiome. A total of 350 14-day-old male Cherry Valley ducks were randomly assigned to five groups receiving diets with 0%, 4%, 6%, 8%, and 10% paper mulberry powder for 42 days. Growth performance, meat quality, serum immunity, and cecal microbial composition were assessed. The results showed no significant differences in average daily feed intake and feed conversion ratio among treatments, with the 6% paper mulberry group showing the highest average daily gain (79.73 g) (*p* < 0.05). Meat quality parameters, including color, drip loss, cooking loss, and shear force, were not significantly affected by paper mulberry powder supplementation, while the 8% paper mulberry group showed the highest pH_24_ value (5.47) (*p* < 0.05). Serum biochemistry revealed increased total protein (G0, G4, G6, G8, and G10: 41.50, 44.47, 45.58, 45.67, and 45.85 g/L, respectively), albumin (G0, G4, G6, G8, and G10: 18.61, 19.56, 20.29, 20.2, and 20.39 g/L, respectively), total cholesterol (G0, G4, G6, G8, and G10: 5.31, 4.96, 5.37, 5.53, and 5.59 mmol/L, respectively), and high-density lipoprotein cholesterol (HDL) in ducks fed 6%, 8%, and 10% paper mulberry powder, with lower alanine aminotransferase (ALT), aspartate aminotransferase (AST), and total bilirubin (TBI) in the 8% and 10% groups (*p* < 0.05). Cecal microbial diversity was enhanced with paper mulberry powder, particularly in the 6% group, which showed increased Bacteroides abundance (*p* < 0.05). Supplementing duck diets with 6% paper mulberry powder increased average daily gain, without adversely affecting meat quality and health, suggesting its potential as a sustainable feed ingredient in the duck meat industry.

## Simple Summary

In the duck meat industry, high feed costs represent a significant challenge. This study investigated whether paper mulberry powder could serve as a viable supplement for duck feed. To this end, Cherry Valley ducks were divided into groups and fed diets containing varying proportions of paper mulberry powder. The results indicated that the inclusion of 6% paper mulberry powder improved duck growth performance without adversely affecting meat quality. Furthermore, the powder appeared to promote duck health, with concentrations of up to 10% showing no negative effects. This research is of considerable importance as it provides duck farmers with an alternative feed option. The use of paper mulberry powder has the potential to enhance the sustainability and cost-effectiveness of the duck meat industry.

## 1. Introduction

China stands as the global leader in duck meat production, accounting for approximately 70% of the world’s total output [1]. Within the domestic market, duck meat ranks third in consumption frequency, following pork and chicken [2]. Consumer demand for duck meat is on the rise in the domestic market, leading to a notable increase in the population of fast-growing meat ducks. China leads the world by a substantial margin in duck population, with nearly 691 million ducks in 2024, and the total output value of meat ducks was more than 120.307 billion yuan in China [3]. This dominance can be attributed to China’s long-established tradition of duck farming, which is propelled by the consistently high domestic demand for duck meat [4,5]. Feed consumption constitutes the primary cost of duck meat production, accounting for approximately two-thirds of the total production expenses [6]. The rising input costs of corn and soybean meal could slow meat duck industry development [7]. Consequently, the duck-farming industry is facing mounting pressure to incorporate forage and by-products, which are more cost-effective and readily available, into poultry diets in order to develop sustainable and economically viable meat duck systems [8].

The investigation of alternative feeding strategies for ducks constitutes a principal focus, encompassing a variety of feeding methods and the utilization of alternative plant-based ingredients [9]. These efforts are directed toward enhancing production efficiency, improving meat quality, and attaining economic advantages. Ducks possess a well-developed digestive organ called the cecum, which allows them to digest many plant-based roughage [10]. Furthermore, the inclusion of fiber-rich ingredients in duck diets is considered beneficial, as it improves intestinal morphology and promotes growth [11]. Additionally, the supplementation of alfalfa meal has been shown to enhance the microbial composition of the cecum in growing ducks [12]. Despite the implementation of forage substitution strategies, which have reduced the reliance on corn and soybean meal, these efforts remain inadequate to satisfy the escalating demand for meat. Hence, China has issued guidelines designed to expedite the development of a diversified food supply system, thereby ensuring grain security and enhancing its agricultural capabilities [13]. Woody forages are assuming an increasingly significant role as a potential natural feed resource, contributing to the alleviation of feed shortages and the promotion of sustainable development in the poultry industry [14]. The use of woody forage to raise animals has become an economical and practical way to avoid food crises in developing countries [15]. Norway also employs woody plants to produce fermented feed, which is utilized to nourish fish in order to meet the human demand for meat protein [16]. Paper mulberry, a well-known woody forage in Eastern Asia, is utilized as feed to replace alfalfa due to its high protein content (13–18%, based on dry matter), with an acceptable content of fiber (neutral detergent fiber at 55.05%, acid detergent fiber at 25.99%, based on dry matter), and the presence of bioactive and antibacterial compounds (total tannin at 1.69%) [17], that provide additional benefits to animal health and animal products are used in poultry such as chickens and geese [18,19]. Moreover, mulberry can regulate the nutritional metabolism process of various livestock and poultry, improve meat quality, and enhance their disease resistance [20]. However, the influence of paper mulberry powder in meat ducks is still not clear. Therefore, this study systematically compared and analyzed the effects of incorporating five distinct dosages of paper mulberry powder into duck diets on their growth performance, meat quality, serum immunity, and cecal microbial composition. The objective of this research is to provide comprehensive foundational data for the development of highly efficient feed formulations supplemented with paper mulberry powder in the meat duck industry.

## 2. Materials and Methods

### 2.1. Ethics Statement

The Cherry Valley ducks’ feeding experimental protocols and procedures were reviewed and approved by the Animal Care and Use Committee of China Agricultural University (AW22601202-5-1). The experiment was carried out on ducks at a commercial farm (Zhuozhou, China) under standard conditions.

### 2.2. Experimental Design and Animal Rearing

The study involved 350 14-day-old male Cherry Valley broiler ducks, which were randomly divided into five groups, each including five replicates with 14 birds. The ducks were supplied by the Charoen Pokphand Group and had been vaccinated prior to being raised. The G0 group was fed a commercialized basal diet without paper mulberry powder, and the G4, G6, G8, and G10 groups (containing 4%, 6%, 8%, and 10% paper mulberry powder, respectively) had paper mulberry powder added to their diets. Duck diets were formulated based on Chinese agricultural industry standards (nutrient requirements of meat-type duck NY/T 2122-2012) [21], and the experimental diet compositions and nutrients are shown in Table 1. Ducks were reared in cages (0.8 m × 0.8 m × 0.5 m) with perforated plastic flooring. The experimental base was disinfected twice a week, and the breeding facility features a comprehensive air conditioning system, as well as an advanced humidity control system, with a temperature inside the house of 20 °C to 26 °C and humidity of 50 to 60%. During the whole experiment, all ducks had ad libitum access to feed and water using nipple drinkers equally. The rearing of these ducks lasted for 42 d.

### 2.3. Growth Performance and Slaughter Performance

The average daily gain (ADG) (g/duck/d) and average daily feed intake (ADFI) were calculated at 42 d. The feed conversion ratio (FCR) was calculated according to ADG and ADFI. At 42 d of age, two ducks with body weights closest to the replicate mean were selected based on health status and weight consistency to minimize variability. Following the weighing procedure, the ducks were euthanized by cervical dislocation. After slaughtering, the pectoral muscles were immediately separated for meat quality measurements (45 min), with another part stored at 4 °C overnight (24 h). The spleen and bursa of fabricius were also weighed. The specific measurement indicators and methods are as follows:

pH: Pectoral muscle samples were cut at 3 different locations using a scalpel. Then, a pH meter (HANNA HI-9024, Hanna, Limena, Italy) was inserted into each location and the results were recorded and calculated.

Meat color: The brightness (L*), redness (a*), and yellowness (b*) values of the meat were measured at 45 min and 24 h using a portable chromameter (CR-400, Konica Minolta, Tokyo, Japan), with three readings taken as the mean [22].

Drip loss: After slaughter, approximately 10 g of meat was excised from the fixed position of the left pectoral muscle, and the connective tissue on the fleshy surface was carefully removed. The muscle was collected in a plastic bag, maintaining its initial mass, and placed upside down along the direction of muscle fibers in cold storage at 4 °C. After 24 h, it was retrieved, and any surface moisture was gently wiped off using absorbent paper before weighing to determine final quality and calculate drip loss. The drip loss percentage was measured as per the method previously described [23].

Cooking loss: Cooking loss was determined by Cardoso’s method [24]. Briefly, to accurately measure the cooking loss, we carefully weighed the 2.5 cm thick mutton pieces and placed them into the preheated steamer. We then inserted a temperature probe into the center of each sample until it reached 70 °C. Afterward, we removed the samples, drained any surface moisture, and weighed it again to calculate the cooking loss.

Shear force: Three muscle strips were removed from each duck, cut into long strips parallel to the muscle fiber with the length, and then detected by a Warner–Bratzler shear machine [25].

### 2.4. Serum Biochemical Parameters and Immunological Analysis

At the end of the feeding trial, 5 mL of blood was taken from the right vein of each duck and placed into a vacuum anticoagulant tube. After centrifugation at 3000 rpm/min for 20 min, 1 mL of serum was transferred into a 1.5 mL centrifuge tube and stored at −20 °C. Then, serum parameters were determined according to the manufacturer’s instructions (Beijing Jinhaikeyu Biological Technology Development Co., Ltd., Beijing, China).

### 2.5. Analysis of Bacterial Composition in Cecum

After slaughter, the cecal contents of ducks were collected into a sterilized tube, immediately stored in liquid nitrogen, and immediately stored in an ultra-low-temperature freezer (−80 °C) until further analysis. Total DNA was extracted from samples, and the quality and concentration were measured by NanoDrop2000 spectrophotometer (Thermo Fisher Scientific, Waltham, MA, USA). Polymerase chain reaction (PCR) was used to amplify the V3-V4 region of the bacterial 16S rRNA gene using universal primers 806R (5′-GGACTACHVGGGTWTCTAAT-3′) and 338F (5′-ACTCCTACGGGAGGCAGCA-3′). Then, amplicons from different treatments were mixed in equal ratios and sequenced via the 2 × 300 paired-end kit on the Illumina NovaSeq sequencing platform (Illumina, San Diego, CA, USA). The high-throughput gene sequencing of 16S rRNA was carried out by Beijing Biomarker Biotechnology Company (Beijing, China). All raw sequencing reads of 16S rRNA have been deposited into the Sequence Read Archive at the National Center for Biotechnology Information (NCBl) database with BioProject no. PRJNA1266638.

### 2.6. Statistical Analysis

The IBM SPSS 24.0 software (IBM Co., Armonk, NY, USA) was employed to conduct data analysis via one-way ANOVA followed by Duncan’s multiple range test for intergroup comparisons, with statistical significance set at *p* < 0.05. Bacterial sequence data obtained from the duck cecum were analyzed using the BMK Cloud Platform (https://international.biocloud.net/, accessed on 7 April 2024). The analyses included a Venn diagram based on operational taxonomic unit (OTU) levels; a Principal Coordinate Analysis (PCoA) utilizing Bray–Curtis distance metrics; bar plots depicting bacterial relative abundance at both the phylum and genus levels; violin plots of cecal microbiota diversity indices assessed via *T*-tests; and Linear Discriminant Analysis Effect Size (LEfSe) analysis at the genus level, evaluated using Kruskal–Wallis tests.

## 3. Results

### 3.1. Growth Performance and Meat Quality

The growth performance indicators of Cherry Valley ducks were evaluated and are presented in Table 2. No significant differences were observed among the treatments (*p* > 0.05). Notably, the G6 group exhibited the highest average daily gain (ADG) compared to other groups (*p* < 0.05).

The results of the determination of physicochemical features of pectoral muscles under different concentrations in diets of paper mulberry powder are shown in Table 3. There were no significant differences in drip loss, cooking loss, and SF (*p* > 0.05). Moreover, meat color 45 min after slaughter showed no significant differences among all groups with different dosages of paper mulberry powder (*p* > 0.05). Although there was no significant difference in meat pH_45min_, the G8 group maintained a relatively high pH_24h_ value (*p* < 0.05).

### 3.2. Serum Biochemical Parameters and Immune Organ Indices

As shown in Table 4, the serum biochemical parameters of Cherry Valley ducks were detected after feeding them different concentrations of paper mulberry powder for 42 days. Ducks in the G6, G8, and G10 groups exhibited increased serum TP, ALB, TC, and HDL (*p* < 0.05), along with no significant differences in TG, LDL, and DBI (*p* > 0.05). Additionally, the G8 and G10 groups had lower ALT, AST, and TBI compared with the G0 group (*p* < 0.05). As shown in Table 5, supplementation with different concentrations of paper mulberry powder had no significant effects on spleen weight, spleen rate, bursa of fabricius weight, bursa of fabricius rate, IgG, IgA, or IgM in Cherry Valley ducks (*p* > 0.05). However, the supplementation of duck diets with paper mulberry powder significantly increased the level of complement 3 and complement 4 (*p* < 0.05).

### 3.3. Cecal Bacterial Diversity

**Figure 1:**
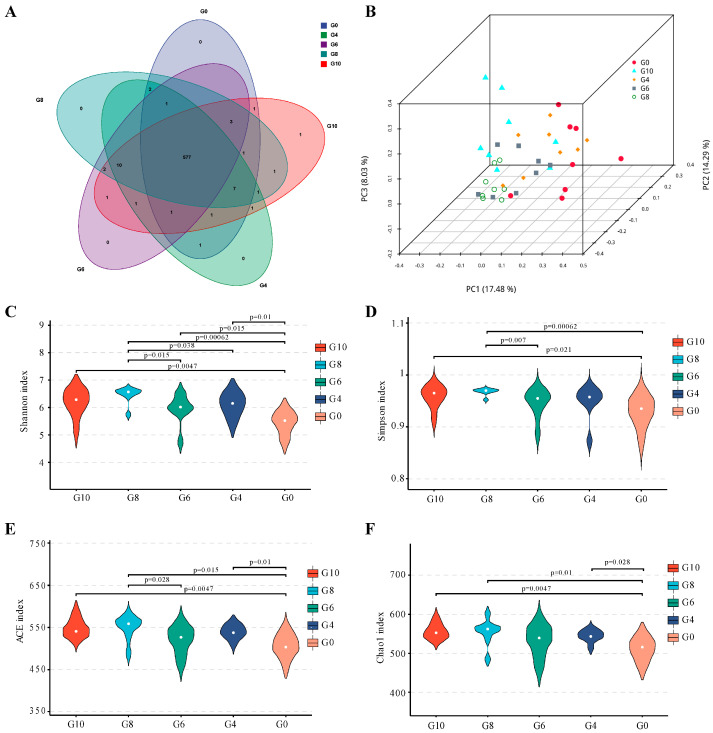
Effects of paper mulberry powder on cecal microbiota diversity index of Cherry Valley ducks. Venn diagram of species numbers (**A**); Principal Coordinate Analysis (PCoA) among groups was plotted based on OTUs (**B**); the values of cecal microbiota diversity indices (**C**–**F**).

A total of 1,470,320 optimized 16S rRNA gene sequences (V3 + V4 region) were obtained, with 613 OTUs detected, while the G0, G4, G6, G8, and G10 groups had 595, 603, 596, 605, and 609 OTUs, respectively (Figure 1A). Principal Coordinate Analysis (PCoA) showed that principal components PC1, PC2, and PC3 explained 17.48%, 14.29%, and 8.03% of the group variation, respectively (Figure 1B). However, there was no clear separation of paper mulberry powder diets from cecal bacterial samples, except that plots from the G8 group were more clustered than other groups. Feeding different concentrations of paper mulberry powder had a significant effect on cecal bacterial diversity indices, as all paper mulberry powder groups showed higher Shannon indices than the G0 group (*p* < 0.05).

**Figure 2:**
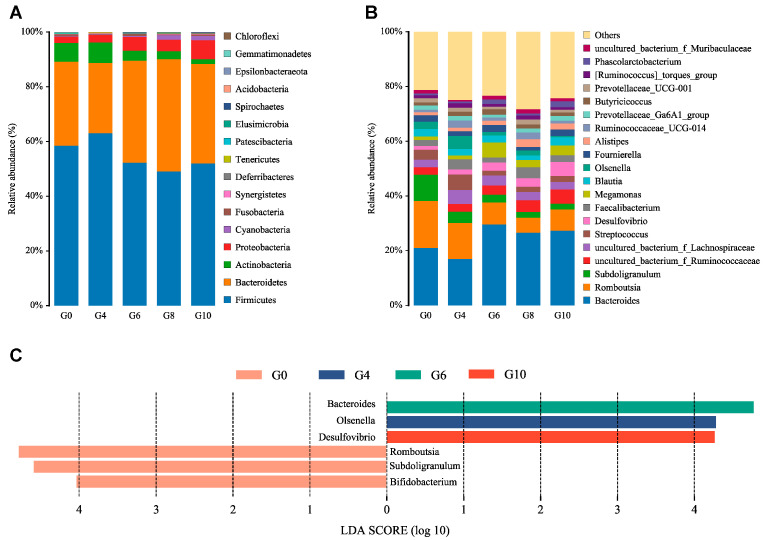
Relative abundance and community taxonomic composition of cecal microbiota at phylum (**A**) and genus (**B**) levels. Linear discriminant analysis (LDA) Effect Size (LEfSe) of the significantly different cecal microbiota (**C**).

Firmicutes, Bacteroidetes, Actinobacteria, and Proteobacteria are the four main phyla in all five groups (Figure 2A). Among these phyla, Bacteroidetes and Proteobacteria showed a trend of increasing with the addition of paper mulberry powder, while Firmicutes and Actinobacteria showed the opposite trend. At the genus level, *Bacteroides* and *Romboutsia* contributed more than 30% of the relative abundance (Figure 2B). *Romboutsia* (17.11%) and *Subdoligranulum* (9.63%) were more abundant in the G0 group than all paper mulberry powder groups, and they could be used as biomarkers in the G0 group via the LEfSe algorithm (Figure 2C). Moreover, *Bacteroides* (29.57%) was more abundant in the G6 group, and *Desulfovibrio* (5.17%) was more abundant in the G10 group (Figure 2C). Different doses of paper mulberry powder in duck diets had significant differences in the bacterial composition of the cecum, and the G6 group significantly increased the abundance of *Bacteroides*.

## 4. Discussion

### 4.1. Growth Performance and Meat Quality

Paper mulberry has mainly been used as herbivore roughage in daily diets, especially for ruminants such as sheep, cattle, and dairy cows [26]. In recent years, as breeding research and animal feeding studies on the varieties of paper mulberry have deepened, it has become evident that this plant not only contributes to enhancing the quality of animal products but also exhibits a broad spectrum of prebiotic functions [27]. Similarly, our previous study showed that paper mulberry could improve the sensory quality and nutritional value of goose meat [18]. So, paper mulberry has also been considered a beneficial feed for poultry diets. The consumption of poultry offers a rich source of high-quality protein and essential nutrients, often exhibiting lower fat content compared to meat derived from alternative animal sources. However, there are limited studies on the incorporation of paper mulberry into duck diets, and the appropriate dosage of paper mulberry in diets remains unclear.

The fact is that ducks are omnivorous birds, with grass constituting a significant component of their natural diet. Previous studies indicated that the inclusion of plant fiber in the diet positively impacts the growth of ducks [28]. Additionally, the supplementation of forage in the diet not only contributes to a reduction in feeding costs but also enhances the overall health of ducks [29]. Suwignyo reported that adding 6% alfalfa hay to the diet had positive effects on the growth of ducks [12]. Paper mulberry is fibrous but has the potential to be used as feed for ducks because of its good palatability, large biomass, and complete nutritional content, including essential amino acids [30]. In the current study, the growth efficiency of ducks was found to be enhanced by adding 6% paper mulberry powder to the diet, which is consistent with previous studies on Guangxi Sanhuang chickens [31]. Meat ducks have a higher tolerance to fiber than broilers, non-significantly affected by feeding Pekin ducks on diets containing fiber up to 12% at two to eight weeks of age. There is scarce scientific literature regarding the supplementation of paper mulberry in duck diets. Our prior research on integrating conformation trees into goose diets revealed that an inclusion rate of 8% did not negatively impact production performance indicators, such as average daily gain and slaughter rate [18]. Although 10% paper mulberry has no adverse impact on duck performance, we recommend a level of 6% considering that elevated crude fiber levels may potentially lead to decreased feed efficiency [32]. Thus, high levels of paper mulberry powder in duck diets should be taken into consideration as they have a limited ability to digest it, with no positive effects.

The meat quality of duck is an increasingly important factor influencing consumer preferences for duck meat products. The pH level of meat serves as a crucial indicator for assessing meat quality, as it directly reflects the acidity of muscle tissue and significantly impacts the processing characteristics of meat. Typically, poultry muscles exhibit pH values (45 min) ranging from 5.6 to 6.5 after slaughter [33]. In alignment with the previous study, the current study showed that the pH value (45 min) in pectoral muscles ranged from 6.12 to 6.27. A previous study reported that high-dose Moringa oleifera leaves reduced the meat color of broilers’ pectoral muscles [34]. Our study showed no statistically significant differences observed in terms of meat color, tenderness, and water-holding capacity among Cherry Valley ducks treated with different dosages of paper mulberry powder. Previous studies have indicated that feed intake or the level of supplement in feed ingredients [35,36,37,38] does not exert any significant influence on meat color and can effectively minimize cost input while having minimal impact on the pH and water-holding capacity of duck meat. In the future, it will be necessary to achieve cross-integration between livestock technology and the agricultural economy in order to conduct more in-depth economic analyses.

### 4.2. Serum Biochemical Parameters and Immune Organ Indices

The serum biochemical parameters play a crucial role in evaluating the metabolism of woody plant feeds in ducks and the overall health condition of duck organisms. In the present study, feeding paper mulberry powder had a significant effect on the serum TP, ALB, ALT, and AST of Cherry Valley ducks. A previous study reported that 5% rosemary leaf supplementation in feed could increase the TP and ALB levels in the serum of broiler chicken [39]. The current study demonstrated comparable findings, wherein the G6, G8, and G10 groups exhibited a significant increase in TP and ALB levels. This suggests that paper mulberry powder facilitates the synthesis and metabolism of liver proteins and enhances the absorption and utilization of tissue proteins [40]. Moreover, the elevated high-density lipoprotein (HDL) levels observed in the serum of groups G6, G8, and G10 suggest that high-dose supplementation of paper mulberry powder may enhance the fat metabolism capability in ducks [41]. AST and ALT are two liver enzymes measured in a serum test to check the health of ducks as high AST and ALT levels are a general sign of a liver problem [42]. Mulberry leaf supplementation in feed decreased ALT and AST levels in the serum of rats and laying hens [43,44], which is in accordance with the G10 group in our study. Collectively, the significantly reduced levels of AST and ALT in the duck serum from the high-dose paper mulberry powder groups, as well as the lack of a significant difference between treatments, suggest that paper mulberry powder does not induce liver damage or toxicity in ducks.

The spleen and bursa of fabricius, as crucial lymphoid organs, play a significant role in the immune system. Therefore, it is of great importance to accurately measure the spleen indices and bursa of fabricius in ducks [45]. As critical immunologically active substances, IgG, IgA, and IgM, together with complements C3 and C4, serve as the key components of humoral immunity that enhance the immune response in ducks [46]. Different levels of paper mulberry powder in diets did not impact the spleen rate or the bursa of fabricius rate but did increase complements C3 and C4 of ducks, suggesting that paper mulberry powder could be used as an immune stimulator to improve the immune function of ducks.

### 4.3. Cecal Bacterial Diversity

The microbiota has garnered considerable attention in recent years due to its profound impact on poultry body health and productivity [47]. To investigate the impact of a paper mulberry powder diet on the intestinal flora of ducks, we conducted an analysis of changes in the cecal microflora through 16S rRNA sequencing. The diversity indices revealed that microbial diversity (Shannon) and abundance (Chao1) were increased by feeding high levels of paper mulberry powder, which indicated a significant effect on the cecal microbiota species of the ducks. The composition of the cecal microbiota plays a pivotal role in the metabolic and digestive processes related to energy utilization in ducks, facilitating the efficient extraction of energy from indigestible dietary fibers through fermentation [11,48].

The current study identified Firmicutes and Bacteroidetes as the predominant phyla in the cecum of Cherry Valley ducks, which is in accordance with previous observations in the cecum of Muscovy ducks [49]. In the current study, the content of Bacteroides in cecum increased with the increase in the supplemental level of paper mulberry powder. The presence of Bacteroides is strongly associated with the digestion of fiber and sugars, and they produce short-chain fatty acids through the fermentation of dietary indigestible polysaccharides and pectin [50]. Moreover, the presence of a substantial amount of Bacteroides can alleviate inflammation through the production of propionic acid [51]. Thus, the higher relative abundance of Bacteroides in the G6 group suggests that the addition of a moderate dosage of paper mulberry powder to the diet could alter the composition of cecum microbiota and modulate cecum health.

Romboutsia belongs to the Firmicutes phylum and is also the dominant genera in the cecal contents of duck [52,53]. Previous research reported that the genus Romboutsia covers a broad range of metabolic capabilities with respect to carbohydrate utilization, the fermentation of single amino acids, anaerobic respiration, and metabolic products [54]. Further, the dietary crude fiber content in the G0 group was comparatively lower than that of the other four groups supplemented with paper mulberry powder. Thus, LEfSe analysis indicated that Romboutsia as biomarkers between the G0 group and the other paper mulberry powder groups may be due to the crude fiber content in duck diets.

Desulfovibrio, a genus belonging to sulfate-reducing bacteria (SRB), is extensively distributed in soil environments, particularly in areas with high metal contamination [55]. This microorganism also colonizes the gastrointestinal tract and contributes significantly to mucosal defense within the digestive system [56]. A study from the Microbiota Protects Against Obesity Project demonstrated that the overgrowth of Desulfovibrio might lead to metabolic diseases in mice [57]. However, prior research has suggested that Desulfovibrio may not always be linked to adverse health effects [58]. Notably, an intriguing study reported that dietary supplementation with Astragalus membranaceus increased the relative abundance of Desulfovibrio in Tibetan sheep, thereby enhancing mucosal defense in the hindgut [56]. Additionally, our previous investigation on lamb rumen also revealed that the paper mulberry treatment exhibited a higher relative abundance of Desulfobulbus, another sulfate-reducing bacterium (SRB) identified in the rumen epithelium [59], which was in accordance with the current study, where paper mulberry powder also increased Desulfovibrio abundance in cecum. Collectively, we hypothesize that the observed increase in serum albumin levels and the decrease in ALT and AST levels within the G10 group may potentially be associated with the significantly higher abundance of Desulfovibrio. This finding implies a possible influence on mucosal defense mechanisms within the gastrointestinal tract of Cherry Valley ducks.

## 5. Conclusions

In conclusion, despite the limited positive impact on feed efficiency in Cherry Valley ducks, incorporating paper mulberry powder at the 6% level could offer a valuable formulation for duck diets. Additionally, the inclusion of a 10% paper mulberry powder supplement in the duck diet did not result in any adverse effects. In future research, it will be essential to further investigate the synergistic effects of paper mulberry powder in combination with other feed ingredients to optimize feed formulations for meat ducks. Additionally, comprehensive studies should be carried out to elucidate the specific mechanisms through which paper mulberry powder influences the gut microbiota of meat ducks, thereby providing a robust theoretical basis for its broader application in meat duck farming.

## Figures and Tables

**Table 1:** Ingredients of experimental diets for Cherry Valley ducks (%, DM basis).

Items	G0	G4	G6	G8	G10
Components (%)					
Corn powder	66.1	64.27	64	62	59.94
Soybean meal	25.95	25.15	24.8	24.3	23.84
Wheat bran	3.25	1.45	-	-	-
Paper mulberry powder	-	4	6	8	10
Soybean oil	1.5	1.93	2	2.5	3.02
Limestone	1.1	1.1	1.1	1.1	1.1
Calcium phosphate	1.2	1.2	1.2	1.2	1.2
NaCl	0.3	0.3	0.3	0.3	0.3
Lysine	0.1	0.1	0.1	0.1	0.1
Methionine	0.2	0.2	0.2	0.2	0.2
Choline chloride	0.1	0.1	0.1	0.1	0.1
Premix ^1^	0.2	0.2	0.2	0.2	0.2
Nutrient contents					
CP, %	18.05	18.05	18.05	18.04	18.05
ME, MJ/kg	2.93	2.92	2.92	2.92	2.92
EE, %	4.17	4.93	5.16	5.82	6.49
CF, %	2.32	3.04	3.38	3.78	4.18
Ca, %	0.8	0.92	0.98	1.04	1.11
P, %	0.61	0.6	0.6	0.6	0.6
Lysine, %	0.91	0.88	0.86	0.84	0.82
Methionine, %	0.28	0.27	0.26	0.26	0.25

^1^ Provided the following per kilogram of diet: vitamin A, 16,000 IU; vitamin B1, 40 mg; vitamin B2, 80 mg; vitamin B5, 40 mg; vitamin B6, 40 mg; vitamin B12, 0.2 mg; vitamin D3 12,000 IU; vitamin E, 800 IU; vitamin K3, 72 mg; biotin, 0.5 mg; folic acid, 16 mg; D-pantothenic acid, 20 mg; nicotinic acid, 20 mg; Cu, 10 mg; Fe, 100 mg; Mn, 40 mg; Zn, 60 mg; I, 80 mg; Se,16 mg; Choline chloride, 500 mg.

**Table 2:** Effect of paper mulberry powder on growth performance of Cherry Valley ducks.

Items	G0	G4	G6	G8	G10	SEM	*p*
ADFI, g	199	192	196	194	200	1.97	0.690
ADG, g	75.22 ^b^	77.1 ^ab^	79.73 ^a^	75.05 ^b^	75.74 ^b^	0.59	0.034
FCR	1.96	1.87	1.86	1.93	1.98	0.02	0.180

Different lowercase letters of peer shoulder notes indicate significant differences (*p* < 0.05), and no letters or identical letters indicate no significant difference (*p* > 0.05).

**Table 3:** Effect of paper mulberry powder on physicochemical features of pectoral muscle of Cherry Valley ducks.

Items	G0	G4	G6	G8	G10	SEM	*p*
pH_45_	6.23	6.27	6.12	6.16	6.12	0.03	0.460
pH_24_	5.34 ^b^	5.32 ^b^	5.38 ^b^	5.47 ^a^	5.35 ^b^	0.02	0.047
a*_45min_	21.15	21.55	21.46	21.25	21.56	0.46	0.990
b*_45min_	7.91	8.77	9.02	9.20	8.39	0.23	0.420
L*_45min_	45.94	45.65	45.28	48.46	44.99	0.61	0.370
a*_24h_	24.48	24.3	24.32	23.75	23.53	0.24	0.630
b*_24h_	10.59	9.37	9.51	9.74	8.46	0.3	0.230
L*_24h_	48.19	46.44	46.47	46.75	46.44	0.48	0.760
Drip loss %	2.22	2.05	2.26	2.46	2.46	0.01	0.640
Cooking loss %	19.2	24.55	21.21	18.32	18.32	0.02	0.870
Shear force N	19.54	18.89	17.2	18.97	18.97	0.61	0.050

Different lowercase letters of peer shoulder notes indicate significant differences (*p* < 0.05), and no letters or identical letters indicate no significant difference (*p* > 0.05).

**Table 4:** Effect of paper mulberry powder on serum biochemical parameters of Cherry Valley ducks.

Items	G0	G4	G6	G8	G10	SEM	*p*
TP (g/L)	41.50 ^b^	44.47 ^a^	45.58 ^a^	45.67 ^a^	45.85 ^a^	0.32	0.028
ALB (g/L)	18.61 ^b^	19.56 ^ab^	20.29 ^a^	20.21 ^a^	20.39 ^a^	0.18	0.039
TC (mmol/L)	5.31 ^a^	4.96 ^b^	5.37 ^a^	5.53 ^a^	5.59 ^a^	0.08	0.026
TG (mmol/L)	2.19	1.75	1.9	1.81	2.17	0.09	0.050
HDL (mmol/L)	1.86 ^c^	2.13 ^b^	2.37 ^a^	2.46 ^a^	2.50 ^a^	0.05	0.017
LDL (mmol/L)	2.58	2.47	2.62	2.7	2.66	0.03	0.050
ALT (U/L)	37.43 ^a^	32.31 ^ab^	38.76 ^a^	33.73 ^ab^	31.26 ^b^	0.57	0.033
AST (U/L)	46.90 ^a^	38.49 ^b^	48.73 ^a^	39.55 ^b^	38.96 ^b^	0.81	0.029
TBI (μmol/L)	13.23 ^a^	12.86 ^a^	13.60 ^a^	11.12 ^b^	12.22 ^ab^	0.24	0.048
DBI (μmol/L)	4.09	4.15	4.66	3.68	4.20	0.09	0.050
α-HBD (U/L)	17.51 ^b^	20.10 ^a^	15.71 ^c^	19.93 ^a^	19.97 ^a^	0.32	0.025
γ-GT (U/L)	5.04	4.98	4.93	5.05	5.16	0.09	0.960

TP, total protein; ALB, albumin; TC, total cholesterol; TG, triglycerides; HDL, high-density lipoprotein cholesterol; LDL, low-density lipoprotein cholesterol; ALT, alanine aminotransferase; TBI, total bilirubin; DBI, direct bilirubin; α-HBD, alpha-hydroxybutyric dehydrogenase; γ-GT, gamma-glutamyl transferase. Different lowercase letters of peer shoulder notes indicate significant differences (*p* < 0.05), and no letters or identical letters indicate no significant difference (*p* > 0.05).

**Table 5:** Effect of paper mulberry powder on immune organ weight and immunoglobulins of Cherry Valley ducks.

Items	G0	G4	G6	G8	G10	SEM	*p*
Spleen rate (%)	0.06	0.07	0.06	0.06	0.07	0.01	0.610
Bursa of fabricius rate (%)	1.08	0.98	1.15	1.21	1.30	0.14	0.220
IgG (mg/mL)	2.5	2.42	2.53	2.44	2.34	0.04	0.770
IgA (mg/mL)	1.06	1.08	1.11	1.03	1.02	0.02	0.570
IgM (mg/mL)	0.81	0.87	0.86	0.77	0.86	0.02	0.190
Complement 3 (mmg/mL)	1.42 ^c^	1.59 ^ab^	1.52 ^b^	1.62 ^a^	1.52 ^b^	0.02	0.048
Complement 4 (mmg/mL)	0.46 ^c^	0.52 ^b^	0.52 ^b^	0.59 ^a^	0.50 ^b^	0.01	0.036

Different lowercase letters of peer shoulder notes indicate significant differences (*p* < 0.05), and no letters or identical letters indicate no significant difference (*p* > 0.05).

## Data Availability

The data presented in this study are available on request from the author. The data are not publicly available due to the privacy policy of the institute.
